# Spatial distribution and temporal trends of leprosy in Uganda, 2012–2016: a retrospective analysis of public health surveillance data

**DOI:** 10.1186/s12879-019-4601-3

**Published:** 2019-11-29

**Authors:** Freda Loy Aceng, Herman-Joseph Kawuma, Robert Majwala, Maureen Lamunu, Alex Riolexus Ario, Frank Mugabe Rwabinumi, Julie R. Harris, Bao-ping Zhu

**Affiliations:** 1 Uganda Public Health Fellowship Program, P.O. Box 7072, Kampala, Uganda; 2 National Tuberculosis and Leprosy Program, Ministry of Health, Kampala, Uganda; 3 German Leprosy and TB Relief Association, Kampala, Uganda; 4 Workforce and Institute Development Branch, Division of Global Health Protection, Center for Global Health, US Centers for Disease Control and Prevention, Atlanta, USA; 5 US Centers for Disease Control and Prevention, Kampala, Uganda; 6 Division of Global Health Protection, Center for Global Health, Centers for Disease Control and Prevention, Atlanta, USA

**Keywords:** Leprosy, Epidemiology, Spatial, Temporal, Trends, Uganda

## Abstract

**Background:**

Leprosy is a neglected disease that poses a significant challenge to public health in Uganda. The disease is endemic in Uganda, with 40% of the districts in the country affected in 2016, when 42 out of 112 districts notified the National Tuberculosis and Leprosy Program (NTLP) of at least one case of leprosy. We determined the spatial and temporal trends of leprosy in Uganda during 2012–2016 to inform control measures.

**Methods:**

We analyzed quarterly leprosy case-finding data, reported from districts to the Uganda National Leprosy Surveillance system (managed by NTLP) during 2012–2016. We calculated new case detection by reporting district and administrative regions of treatment during this period. New case detection was defined as new leprosy cases diagnosed by the Uganda health services divided by regional population; population estimates were based on 2014 census data. We used logistic regression analysis in Epi-Info version 7.2.0 to determine temporal trends. Population estimates were based on 2014 census data. We used QGIS software to draw choropleth maps showing leprosy case detection rates, assumed to approximate the new case detection rates, per 100,000 population.

**Results:**

During 2012–2016, there was 7% annual decrease in reported leprosy cases in Uganda each year (*p* = 0.0001), largely driven by declines in the eastern (14%/year, *p* = 0.0008) and central (11%/year, *p* = 0.03) regions. Declines in reported cases in the western (9%/year, *p* = 0.12) and northern (4%/year, *p* = 0.16) regions were not significant. The combined new case detection rates from 2012 to 2016 for the ten most-affected districts showed that 70% were from the northern region, 20% from the eastern, 10% from the western and 10% from the central regions.

**Conclusion:**

There was a decreasing trend in leprosy new case detection in Uganda during 2012–2016; however, the declining trends were not consistent in all regions. The Northern region consistently identified more leprosy cases compared to the other regions. We recommend evaluation of the leprosy surveillance system to ascertain the leprosy situation.

## Background

Leprosy, also known as Hansen’s disease, is a chronic bacterial infection caused by *Mycobacterium leprae.* The disease may affect the nerves, skin, eyes, and nasal mucosa; if left untreated, nerve damage may cause paralysis of hands and feet and disfigurement [1]. Transmission of leprosy occurs through direct and indirect contact with infectious sources [1–4]. However, due to the slow-growing nature of the bacteria and the long incubation period, it may be difficult to determine the infection source [5]. Leprosy diagnosis is based on clinical presentation and confirmed by skin or nerve biopsy and acid-fast staining [1, 6]. Leprosy is treated by prolonged multidrug therapy involving a combination of antibiotics such as dapsone, rifampicin, and clofazimine [1, 7, 8].

Leprosy is one of the most-neglected diseases and most often affects the poorest populations [9, 10]. In 2016 the WHO reported a global new case detection rate of 2.9 per 100,000 population and a prevalence rate of 0.27 per 10,000 population [11, 12]. The World Health Organization (WHO) developed a strategy “Global Leprosy Strategy 2016-2020: accelerating towards a Leprosy-free world”, aimed at reducing the new leprosy diagnosis rate to fewer than one per million, and eliminating permanent disabilities especially among children affected by the disease in endemic countries [12, 13]. To achieve these goals, early detection through surveillance, and diagnosis and treatment are paramount [14, 15].

Leprosy has historically posed a public health challenge in Uganda [14]. The disease is currently endemic; in 2016, 42 out of 112 districts reported at least one case of leprosy [14]. In areas of leprosy endemicity, spatial clustering of patients is frequent [16]. During 2016 in Uganda, the leprosy prevalence was 0.07 cases per 10,000; the National Tuberculosis and Leprosy Program (NTLP) in Uganda set a goal to reduce leprosy prevalence by 30%, to 0.05 cases per 10,000, by 2020 [14].

The NTLP has implemented interventions to reduce the burden, such as community skin camps (community outreaches with free leprosy screening), refresher training of health workers, and contact tracing visits, particularly in areas endemic for leprosy [14]. Despite these efforts, new cases are still being reported in Uganda. During 2015/2016, 217 new leprosy cases were reported, a new case detection rate of 0.6/100,000. Of the 217 cases, 6% were in children < 15 years old and of those 69% were from the high burden region (Northern), indicating relatively recent spread at the community level [14]. Approximately 27% of leprosy patients had Grade 2 disabilities (severe visual impairment or visible deformities) at diagnosis, indicating a substantial delay in case detection [12, 14]. We determined the spatial distribution of leprosy in Uganda, and assessed temporal trends of leprosy diagnoses during 2012–2016 to inform control measures.

## Methods

### Study area

**Fig. 1:**
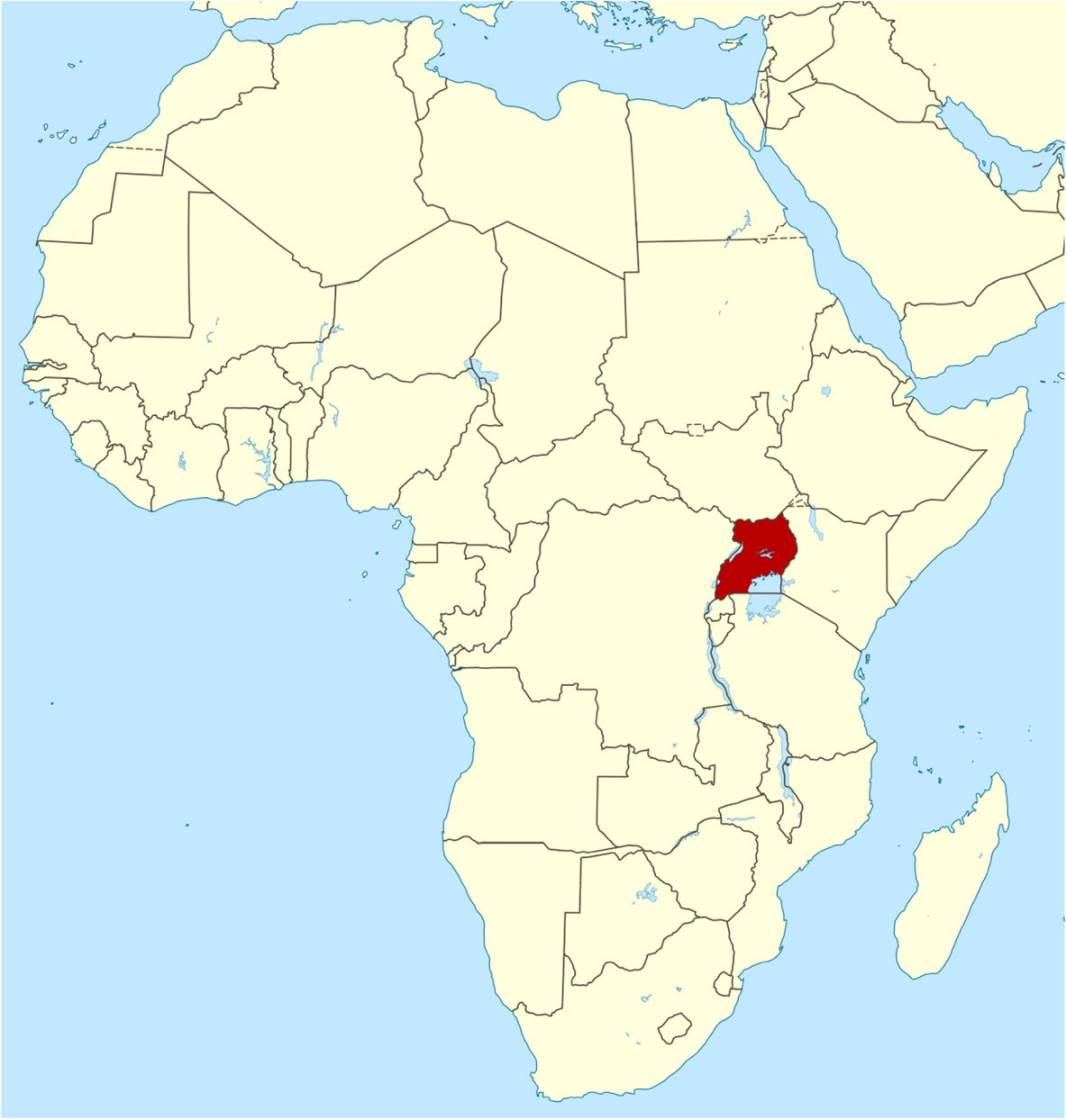
Map of Africa showing Uganda. The map was taken from https://www.mapsland.com/. Their copyright policy states that all the material (articles and images, unless otherwise specified) are published under the Creative Commons Attribution-ShareAlike 3.0 Licence

Uganda (Fig. 1) is divided into four administrative regions (Central, Western, Northern and Eastern), which are further subdivided into 112 districts [17]. Uganda has 155 hospitals, of which two are National Referral Hospitals, 14 are Regional Referral Hospitals (RRHs), and 139 are General Hospitals (GHs). The hospitals receive suspected leprosy cases from health facilities called Health Centers II, III and IV (MOH, 2015). Six health facilities, five GHs and one HC III, currently have leprosy treatment centers. These include two facilities in Eastern Uganda, three in Northern Uganda, and one in Western Uganda.

### Description of the leprosy surveillance system in Uganda

In Uganda Ministry of Health, leprosy is managed under the National Tuberculosis and Leprosy Programme (NTLP); a Central Unit of the NTLP is responsible for policy formulation, planning, resource mobilization and monitoring. There are focal persons for the combination at regional and district levels. Programme implementation especially the patient care activities are integrated into the primary health care system; staff responsible for leprosy treatment and care do so as part of other responsibilities in the health facilities where they are located.

**Fig. 2:**
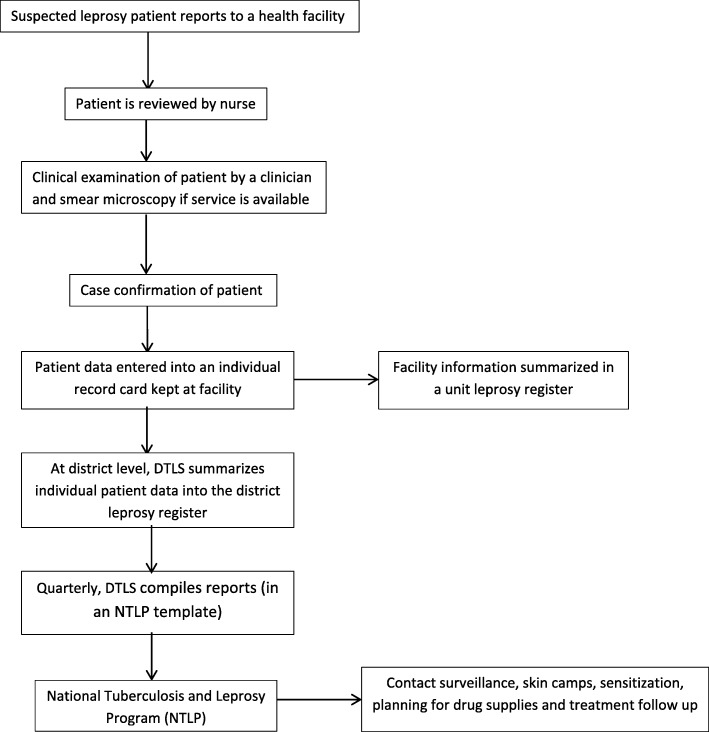
The Uganda Leprosy surveillance

At the facility level, patients seeking care for leprosy and other conditions have information recorded on an individual record card. Patients with suspected leprosy have clinical data summarized in a unit leprosy register, used at the district level by the District Tuberculosis Leprosy Supervisor (DTLS) to summarize all patient data into a district leprosy register. On a quarterly basis, the DTLS compiles a quarterly report using a standard reporting template developed by the NTLP. The report includes summaries of the numbers and characteristics of new cases registered, and the number of patients still on treatment at the end of the quarter. The NTLP uses this information to plan and conduct contact surveillance, skin camps, sensitization, planning for drug supplies, and treatment follow up (Fig. 2). There were 6–8 skin camps per year. There are approximately 250 staff involved in leprosy work in its different perspectives: clinical, administrative, social work and rehabilitation services. There is at least one district level focal person in each of 112 districts. In the leprosy surveillance system, leprosy cases are notified by the districts that diagnosed them rather than by their districts of residence; however, leprosy cases that go to referral centers are mostly from the same region as the referral center.

#### Study design

We conducted a retrospective analysis of routinely-generated program data reported through the NTLP leprosy surveillance system.

#### Study population

The study population comprised cases of leprosy diagnosed by the health services between 2012 and 2016.

### Leprosy case definition

A case of leprosy (as defined by NTLP guidelines) was defined as a person with one or more of: reddish or hypo-pigmented skin lesion(s) with definite loss of sensation; damage to the peripheral nerves, as demonstrated by nerve enlargement and loss of sensation and or weakness of the muscles in parts supplied by these nerves; skin smear-positive for acid-fast bacilli [14]. Most leprosy cases are diagnosed using clinical findings alone [18].

#### Source of data and collection procedure

We abstracted data from the NTLP leprosy surveillance database. All the data generated between 2012 and 2016 were considered for the study. We abstracted data on leprosy cases by district that reported them and administrative regions.

### Data management and analysis

We entered data in Excel spreadsheets and exported to Epi Info version 7.2.0 (US Centers for Disease Control and Prevention) for analysis. We used logistic regression to determine the temporal trends, and used QGIS (Quantum Geographic Information System) software to determine the spatial trends. We calculated new case detection rates using the new cases per district and individual district populations and drew choropleth maps for Uganda showing the new case detection rates of leprosy per 100,000. Population estimates were calculated from the 2014 National Population and Housing Census, and a national growth rate of 3% was used to estimate the yearly populations [17].

## Results

**Fig. 3:**
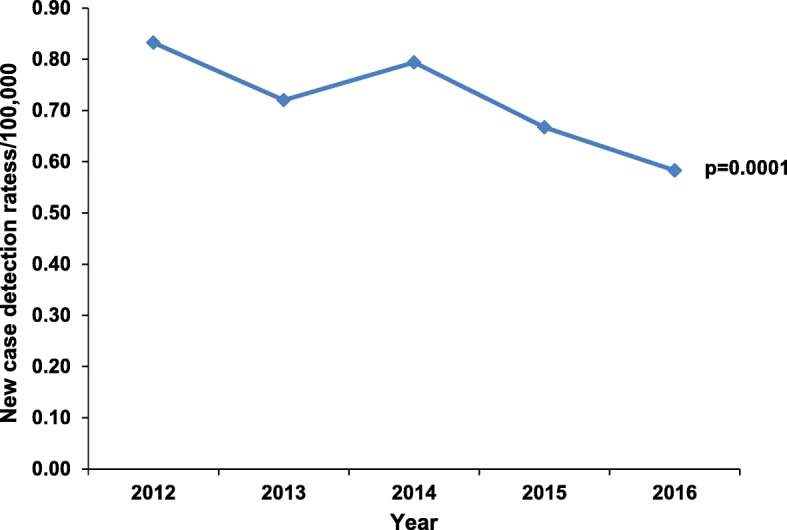
Leprosy new case detection rates, Uganda, 2012-2016 *p = *p*-value for trend

**Fig. 4:**
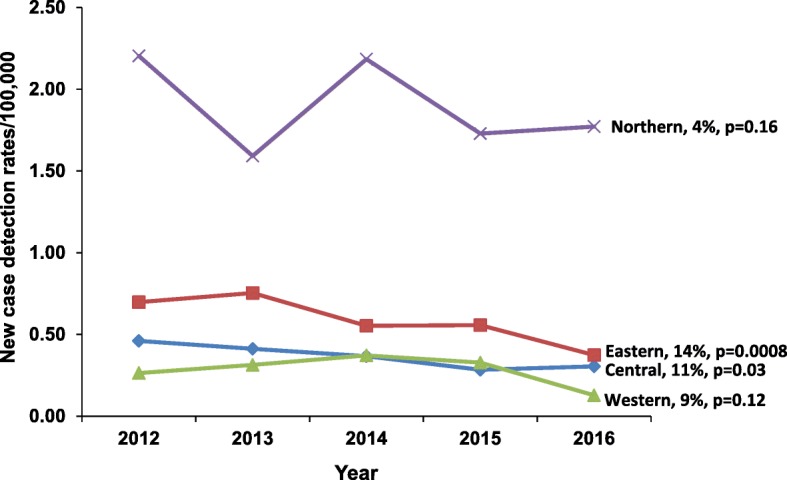
Regional trends of leprosy new case detection rates, Uganda, 2012-2016 *p = *p*-value for trend

Over the entire study period, 1240 new cases of leprosy were reported. The leprosy indicators for Uganda are shown in Table 1. There was a 7% annual decrease in the number of new leprosy cases from 2012 (271 cases) to 2016 (214 cases) (*p*-value for trend = 0.0001) (Fig. 3). The Eastern region showed a decline of 14% each year over the same time period (*p* = 0.0008), and the Central region showed a decrease of 11% (*p* = 0.03). Changes in new leprosy cases reported in the Western and Northern regions were not statistically significant (Fig. 4).

**Table 1:** Leprosy indicators for Uganda

Indicators for Uganda	Location	2012	2013	2014	2015	2016
The number of new leprosy cases	National level	271	242	275	238	214
The New Case Detection Rate/100,000		0.83	0.72	0.79	0.67	0.58
The proportion of children <15		9%	5%	6%	5%	6%
The number and proportion of females		48%	45%	50%	43%	47%
The MB proportion of new cases		86%	84%	89%	90%	86%
The proportion of new cases presenting with grade 2 disabilities	National level	24%	33%	28%	25%	22%
	Northern region	17%	28%	20%	16%	12%

**Fig. 5:**
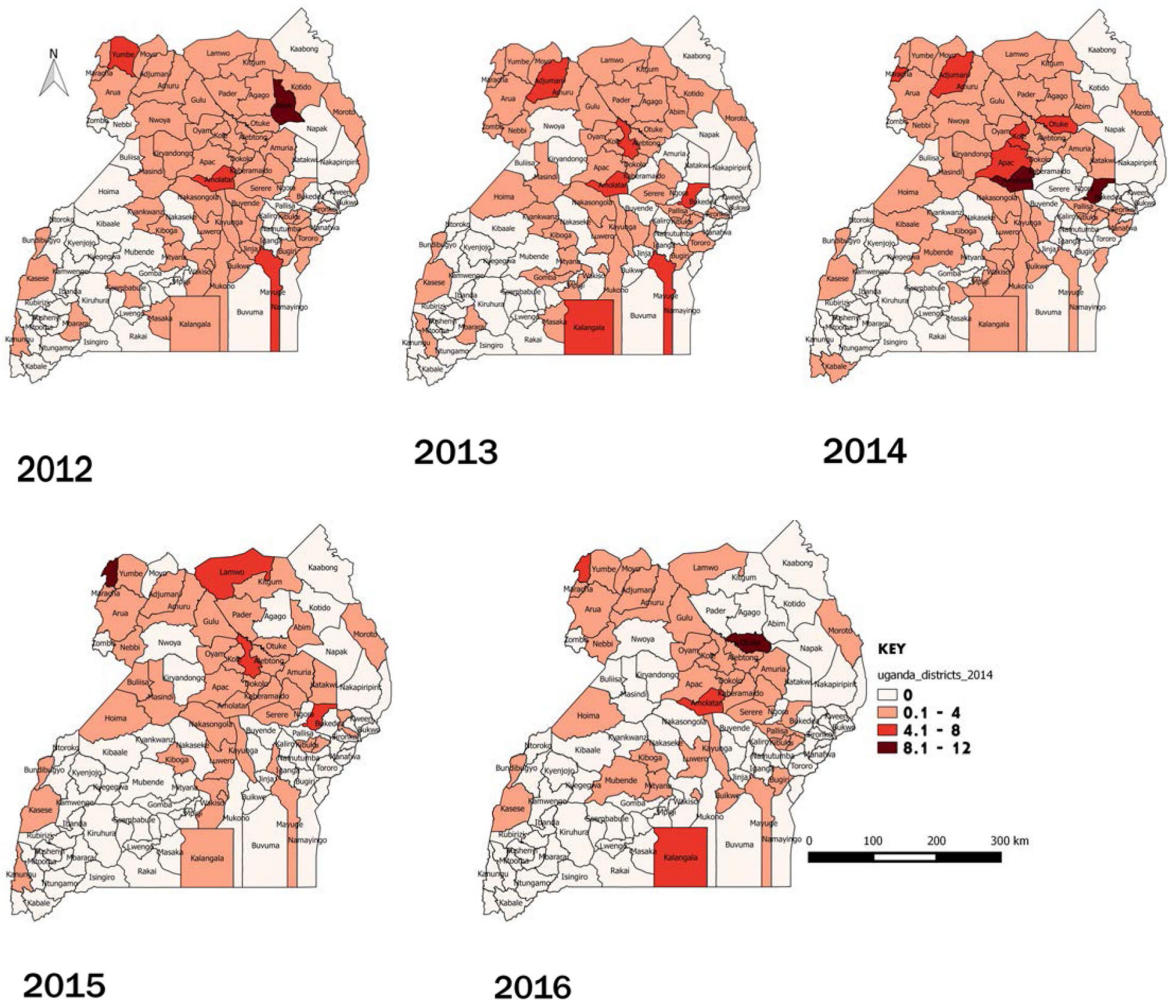
Leprosy new case detection rates per 100,000, Uganda districts, 2012-2016

Of the 10 most-affected districts during 2012–2016, 60% were from the northern region, 20% were from the eastern region, 10% were from the western region and 10% were from the central region (Fig. 5).

## Discussion

We found that there was a decrease in overall new reported cases of leprosy in Uganda during 2012–2016. The decline could be due to a stable, longstanding control program, the presence of at least one trained focal person in every district, and financial stability from a single implementing partner funding the program (in Uganda’s case, the German Leprosy Relief Association). It may also be related to overall secular trends in leprosy detection: according to the WHO’s Global leprosy strategy 2016–2020, the global case detection rate, defined as the notification rate per 100,000 population, seems to be declining slowly. However, in many areas it has remained static, and in a few places increased. Changes in detection rates happen slowly, over decades, due to the long incubation period of the disease. They are related to factors such as BCG vaccination coverage, socioeconomic development, and good leprosy programs’ practices, especially early diagnosis [19].

However, the decline may be due to low-level knowledge and skill among health care provider and limited access to health facilities. Thus, reported cases in NTLP leprosy surveillance database might not actually represent the actual situation on the ground. This is due to weakening of the intermediate support system (at regional and district levels) and the decreased coverage of data collection tools in current use. The system picks up mostly obvious multibacillary cases and with established disability.

The central region has experienced a rapid poverty reduction between 2006 and 2013 [20] that might result into fewer persons in the community becoming infected. The decreasing temporal trends might also be attributed to socio-economic factors such as improved nutrition, hygiene, and awareness [16]. While the long incubation period of leprosy makes it difficult to gauge exactly when individual infections were acquired, assuming an unchanging mean incubation period over time, we may expect that infections in general are indeed on the decline. However, there are areas in Uganda that still have new cases being reported, and these areas may require extra attention in order to improve the diagnosis and management of the patients.

Cases were reported from all four regions over the entire evaluation period. The spatial trends showed that the Northern districts consistently had higher number of leprosy cases and higher new case detection rates per 100,000 populations. However, this may also be due to the higher number of treatment centers available in the Northern region, compared with other regions. It is also possible that this region may have a better surveillance system that quickly reports the leprosy cases identified.

We noted from the maps that there was apparent clustering in endemic regions. This situation was similar in South India [16], where there was clustering in one of the regions. Leprosy is highly endemic in the Northern region [14] and since it is of a chronic nature, this results into slower temporal changes [16]. In addition, due to the endemic nature of leprosy in the Northern region, the community awareness is also higher and the stigma associated with reporting is lower. This may result in better health-seeking behavior and more cases being identified. Both the ongoing infections and the higher rates of healthcare-seeking in the Northern Region are supported by existing data; new cases in this region rarely have Grade 2 disabilities indicative of advanced disease [14]. In 2016, the overall grade 2 disability rate for Uganda was 22 and 12% for the Northern Region as shown in Table 1. In addition, the skin camps are conducted in high burden districts in the Northern region so as to identify more cases.

The major limitation with our study was the use of program data, which recorded only the notifying district that diagnosed the leprosy cases rather than their district of residence. There could be an overrepresentation of the leprosy burden in districts with leprosy treatment centers or, more importantly, an underrepresentation from areas without leprosy treatment centers. The heterogeneity in the distribution of new case detection rates of leprosy in the various districts of Uganda may be due to differing qualities in the district surveillance systems. In addition, some new cases that were identified in the leprosy referral centers could have been missed by their respective districts. However, this is unlikely to alter the temporal and spatial trends greatly since most of the people seek leprosy services from within their regions. It is also possible that there are leprosy cases that are not identified in or notified through the program, making the true burden estimates of leprosy challenging. Although we used the minimum recommended number of years for studying leprosy trends, the best approach is by analyzing data of ten years or more [21] as variations from one year to the next might be hard to explain due to operational factors that are not always easy to determine in retrospect. The secondary data at national level which were used in this study were characterized by inconsistency in recording and reporting and changing numbers and demarcations of districts which resulted in challenges with analysis of data at regional level. Assuring the completeness of the data would have required time and other resources to look for and review records at lower levels.

## Conclusions

In summary, the leprosy rates in Uganda are continuing to decline. The Northern region consistently identified more leprosy cases compared to the other regions. We recommend evaluation of the leprosy surveillance system to ascertain the leprosy situation. There should be more in-depth analysis of new case detection especially in the so called high-burden areas at sub-district level. In particular, the district of residence of all cases should be identified. This is important as the location of the referral centers was in the first place determined by having high prevalence of leprosy in those areas at that time.

## Data Availability

The datasets used and analyzed during this study are available from the corresponding author on reasonable request.

## Abbreviations

CDC: Centers for Disease Control and Prevention
DTLS: District Tuberculosis and Leprosy Supervisor
GHs: General Hospitals
MDT: Multidrug therapy
NTLP: National Tuberculosis and Leprosy Program
PFP: Private for-profit
PNFP: Private not-for-profit
QGIS: Quantum Geographic Information System
RRHs: Regional Referral Hospitals
WHO: World Health Organization
